# Nucleolin represses transcription of the androgen receptor gene through a G-quadruplex

**DOI:** 10.18632/oncotarget.27589

**Published:** 2020-05-12

**Authors:** Cindy K. Miranti, Sara Moore, Yongeun Kim, Venkateshwar Reddy Chappeta, Kui Wu, Biswanath De, Vijay Gokhale, Laurence H. Hurley, Elsa M. Reyes-Reyes

**Affiliations:** ^1^ University of Arizona Cancer Center, University of Arizona, Tucson, AZ 85724, USA; ^2^ Department of Cellular and Molecular Medicine, Tucson, AZ 85724, USA; ^3^ University of Arizona College of Medicine, Division of Pulmonary, Allergy, Critical Care, and Sleep Medicine, Tucson, AZ 85724, USA; ^4^ College of Pharmacy, University of Arizona, Tucson, AZ 85724, USA; ^5^ BIO5 Institute, University of Arizona, Tucson, AZ 85724, USA

**Keywords:** prostate cancer, androgen receptor, nucleolin, gene repression, G-quadruplex

## Abstract

The androgen receptor (AR) is a major driver of prostate cancer development and progression. Men who develop advanced prostate cancer often have long-term cancer control when treated with androgen-deprivation therapies (ADT). Still, their disease inevitably becomes resistant to ADT and progresses to castration-resistant prostate cancer (CRPC). ADT involves potent competitive AR antagonists and androgen synthesis inhibitors. Resistance to these types of treatments emerges, primarily through the maintenance of AR signaling by ligand-independent activation mechanisms. There is a need to find better ways to block AR to overcome CRPC. In the findings reported here, we demonstrate that the nuclear scaffold protein, nucleolin (NCL), suppresses the expression of AR. NCL binds to a G-rich region in the AR promoter that forms a G-quadruplex (G4) structure. Binding of NCL to this G4-element is required for NCL to suppress AR expression, specifically in AR-expressing tumor cells. Compounds that stabilize G4 structures require NCL to associate with the G4-element of the *AR* promoter in order to decrease AR expression. A newly discovered G4 compound that suppresses AR expression demonstrates selective killing of AR-expressing tumor cells, including CRPC lines. Our findings raise the significant possibility that G4-stabilizing drugs can be used to increase NCL transcriptional repressor activity to block AR expression in prostate cancer. Our studies contribute to a clearer understanding of the mechanisms that control AR expression, which could be exploited to overcome CRPC.

## INTRODUCTION

Prostate cancer (PCa) is the second leading cause of cancer-related deaths in men [1]. Androgen receptor (AR) drives prostate cancer by regulating specific programs critical for tumor survival and growth. Androgen deprivation therapy (ADT) is the mainstay treatment for prostate cancer patients with advanced disease. ADT suppresses the function of AR by depriving it of its ligand androgen, either by suppressing androgen biosynthesis or as competitive antagonists [2]. Despite the clinical successes of new ADT agents, such as the androgen biosynthesis inhibitor abiraterone and AR antagonist enzalutamide, PCa patients still become unresponsive [3–6]. Cancer recurs after ADT within 1–3 years as castration-resistant prostate cancer (CRPC) [7]. The aberrant reactivation of AR is often independent of its ligands, and continued down-stream signaling is the main culprit of ADT resistance and a vexing therapeutic problem.

AR signaling in CRPC persists by multiple mechanisms such as amplification of the *AR* gene, *AR* gain of function mutations, induction of other signaling pathways that activate AR, and *AR* splice variants that display constitutive activity in the absence of ligand binding. Most CRPC cases have an increase in AR protein production [8, 9]. Extensive research indicates the ablation of AR expression, as opposed to simply blocking its activity, offers a possible pathway to a favorable treatment for CRPC. However, the molecular mechanisms that regulate *AR* expression are poorly understood. Hence, there is a critical need to define novel mechanisms that regulate *AR* transcription and identify targets that block *AR* expression to develop new ways to overcome resistance to current therapies for patients with CRPC.

The gene for *AR* is located on the X chromosome (q11–12) and expresses a 110-kDa protein of 919 amino acids encoded by eight exons [10, 11]. The *AR* gene has two transcription initiation sites located at 1116 base pairs (bp) (TIS I), and 1127 bp (TIS II) upstream of the *AR* translation start codon. Tilley et al. identified a cis-nucleotide guanine (G)-rich sequence within the *AR* gene promoter located close to the Specific Protein 1 (Sp1) motif, which is conserved among humans, rats, and mice [12]. This G-rich region was reported to be a critical regulatory cis-acting element of the transcriptional activity of *AR* [13, 14]. The double-strand conformation of the G-rich region can bind nuclear proteins to activate *AR* transcription. A single-strand structure of this G-rich region, however, was reported to induce the binding of unidentified proteins that interfere with assembly of the transcriptional initiation complex at the *AR* promoter [12, 14, 15]. These studies defined the G-rich region in the *AR* gene as an essential regulatory element.

Certain guanine-rich sequences in the presence of monovalent cations generate G-quartet stacks to form nucleic acid secondary structures called G-quadruplexes (G4). G4s have been found in the promoters of a wide range of genes associated with oncogenesis, such as *MYC, KRAS, VEGF, BCL-2, PDGFR,* and *HIF-1α*. Compounds have been identified, which can specifically stabilize G4 structures [16] and suppress the transcription of genes that contain a G-rich region with the potential to form G4s [17]. Mitchell et al. showed that the G-rich region of *AR* can form parallel G4 structures [18]. Moreover, some G4-stabilizing agents can repress *AR* expression and cell growth of prostate cancer cell lines [18, 19].

Nucleolin (NCL) is an RNA-binding protein that has multiples roles in ribosome biogenesis, transcription, DNA and RNA metabolism, DNA repair, and apoptosis [20, 21]. Although more than 90% of NCL is localized in the nucleolus, it is also present in other cellular compartments such as the nucleoplasm, cytoplasm, and cell surface. NCL regulates transcription through different mechanisms. In the nucleolus, NCL positively regulates rRNA transcription by two mechanisms, enhancing the transcriptional activity of RNA polymerase I [22] and promoting chromatin decondensation by collaborating with chromatin remodelers [23–25]. In the nucleus, NCL regulates Pol II-based transcription of some genes by binding to G4-structures localized in the promoters. NCL binding to G4 can either activate or repress transcription. NCL suppresses *c-Myc* [26] but increases *VEGF* and *NPGPx* transcription via G4 structures [27, 28].

The specific molecular mechanisms for how the G4-element within the *AR* promoter regulates its transcription remain unclear. In the study reported here, we demonstrate that the binding of the nuclear scaffold protein, NCL, at the G4-element of the *AR* promoter is essential to suppress AR expression, and G4-stabilizing drugs that suppress AR require NCL.

## RESULTS

### Nucleolin is associated with the G4-element in the AR gene promoter

**Figure 1:**
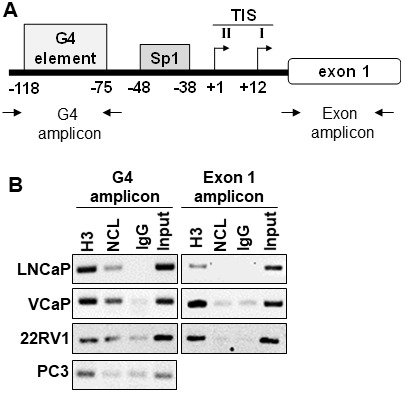
Nucleolin associates with the G4-element within the AR promoter. (**A**) Schematic location of the primers encompassing the G4 and Exon 1 regions within the *AR* gene. (**B**) ChIP assay. Proteins were cross-linked to the DNA in prostate cancer cells with formaldehyde. Chromatin was sheared, and protein-DNA complexes were immunoprecipitated with antibodies against NCL (NCL). Isotype IgG (IgG) and Histone 3 (H3) antibodies served as negative and positive controls, respectively. Input represents 2% of total cross-linked chromatin before immunoprecipitation. Retrieved DNA was amplified using primers to encompass G4 and Exon1 regions. Data are representative of three independent experiments.

Previous studies reported that the G-rich region in the *AR* gene promoter forms a parallel G4 structure with a long central loop of 11 or 13 base pairs [18, 29]. NCL binds with a high affinity to G4s with long loops [30–32]. To elucidate the molecular mechanism of how the *AR* G4-element regulates AR expression, we determined whether NCL binds to the G4-element of the *AR* promoter using chromatin immunoprecipitation (ChIP) in prostate cell lines that show similar NCL protein expression (Supplementary Figure 1). Following ChIP for NCL, PCR amplification of the *AR* promoter region containing the G4-element was compared to a non-G4 region in exon 1 (Figure 1A). ChIP revealed that NCL binds to the region of the *AR* promoter containing the G4-element in androgen-dependent (LNCaP/VCaP) and CRPC (22Rv1) AR-expressing tumor cells, but not in AR-negative tumor cells (PC3) (Figure 1B). NCL did not bind to the non-G4 region in exon 1. Histone H3, but not negative IgG control, was present at both sites, G4-element and non-G4 region in exon 1, in all the cell lines. These data show that NCL is constitutively associated with the *AR* promoter G4-element specifically in prostate cancer cells that express AR, and suggest that NCL plays a role in regulating the *AR* promoter through its G4-element.

### Nucleolin suppresses AR expression by binding to the G4-element

**Figure 2:**
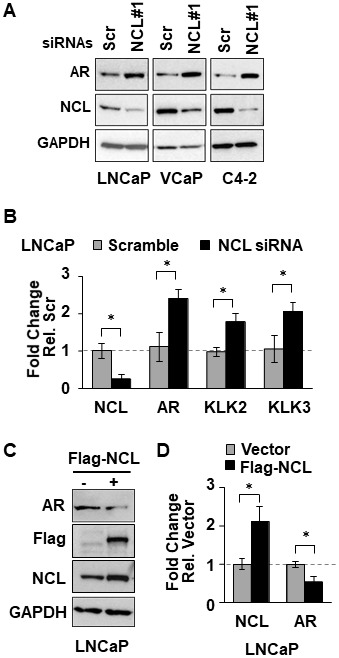
NCL suppresses AR expression. (**A**, **B**) Indicated prostate cancer cell lines were transfected with NCL (NCL#1) or scrambled (Scr, control) siRNAs. (**C**, **D**) LNCaP cells transfected with Flag-NCL cDNA or empty vector. (A, C) Cell lysates were analyzed for expression of AR, NCL, Flag-tagged NCL, and GAPDH by immunoblotting. (B, D) Extracted RNA was analyzed for expression of (B) NCL, AR, KLK2, and KLK3 (PSA), or (D) NCL and AR by RT-qPCR. Values are means ± SD; *p <* 0.05 (^*^); *n =* 3.

NCL is a nuclear protein that has multiples roles in ribosome biogenesis, transcription, DNA and RNA metabolism, and DNA repair within the nucleus [20, 21]. NCL can exert transcriptional regulatory activity through high-affinity binding to G4-elements [26–28, 33]. Therefore, NCL association at the *AR* G4-element could regulate *AR* promoter activity. To test this hypothesis, we measured the effect of manipulating NCL expression on AR expression. NCL expression was knocked-down by 80% with two siRNAs in three human AR-positive prostate tumor cell lines, LNCaP, VCaP, and C4-2. Suppression of NCL expression increased the levels of both AR protein (Figure 2A and Supplementary Figure 2) and mRNA (Figure 2B) relative to a scrambled siRNA control independently of the level of AR expression in these PCa cells (Supplementary Figure 1). NCL knockdown also increased the mRNA levels of two AR target genes, *KLK2* and *KLK3* (PSA) (Figure 2B). Conversely, NCL overexpression reduced AR protein and mRNA relative to the vector control (Figure 2C and Figure 2D). These data indicate that NCL negatively regulates *AR* expression.

**Figure 3:**
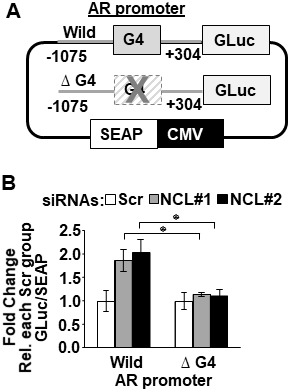
Nucleolin-mediated suppression of *AR* promoter activity depends on the *AR* G4-element. (**A**) Schematic representation of dual-luciferase/SEAP reporter, containing the *AR* promoter (–1075 to + 304) with the G4 sequence intact (Wild) or deleted (Δ G4). (**B**) LNCaP cells stably expressing the AR promoter dual-luciferase/SEAP reporter were transfected with scrambled (Scr) or two NCL siRNAs (#1, #2). Luciferase activity was normalized to G-Luc/SEAP ratio and expressed as fold change relative to scrambled (Scr) control. Values are means ± SD; *p <* 0.05 (^*^); *n =* 3.

To measure the dependency of NCL-mediated *AR* suppression on the *AR* G4-element, we generated stable LNCaP cell lines expressing a dual reporter. In the transcriptional reporter, the Gaussia luciferase is driven by either a wild type or a mutant *AR* promoter lacking the G4 element (ΔG4), and the secreted alkaline phosphatase (SEAP) is driven by a constitutive promoter (Figure 3A). The deletion of the G4 DNA segment in the *AR* promoter only decreased luciferase reporter activity by 40% compared to the wild type *AR* promoter (Supplementary Figure 3A). Full serum increased luciferase activity 2-fold in cells expressing the ΔG4 *AR* promoter reporter compared to charcoal-stripped low serum (Supplementary Figure 3B). These results indicate that even when the G4-element is deleted, this mutant *AR* promoter is still capable of sustaining *AR* transcription.

NCL expression was then knocked down in these reporter cells using two NCL-specific or scrambled siRNA sequences. Figure 3B shows that the cells expressing the wild type *AR* promoter exhibited a significant increase in luciferase activity after suppressing NCL expression (*p <* 0.05) compared to scrambled siRNA transfected cells. However, the deletion of the G4 element prevented luciferase up-regulation in the absence of NCL (Figure 3B and Supplementary Figure 3C). These data, combined with the ChIP data in Figure 1, indicate NCL acts as a negative *AR* transcriptional regulator by binding to the *AR* G4-element.

### G4-binding agents recruit NCL to the G4-element to suppress AR expression

**Figure 4:**
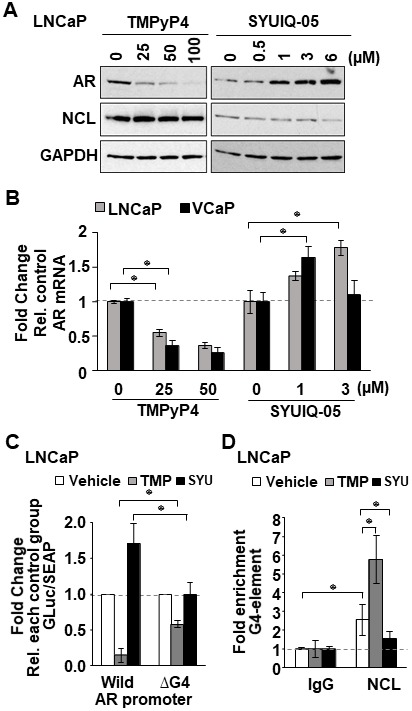
G4-stabilizing agents influence NCL association with the G4 in the *AR* promoter. (**A**, **B**) Indicated tumor cell lines treated with increasing concentrations of two known G4-binding agents, TMPyP4 and SYUIQ-05. (A) AR, NCL, and GAPDH expression measured by immunoblotting. (B) *AR* mRNA analyzed by qRT-PCR. (**C**) Relative luciferase in cells expressing the *AR* G4 (Wild) or deleted (ΔG4) reporter, treated with 25 μM TMPyP4 (TMP) or 3 μM SYUIQ-05 (SYU). Luciferase activity was normalized to G-Luc/SEAP ratio and expressed as fold change relative to vehicle (DMSO). (**D**) ChIP of NCL on *AR* G4 in the absence or presence of 25 μM TMP or 3 μM SYU. Negative (IgG) control. Fold enrichment relative to IgG. Values are means ± SD; *p* < 0.05 (^*^); *n =* 3.

Compounds that bind to G4 structures can regulate the transcription of genes that contain G4-elements in their promoters [17]. To test whether G4-binding compounds might suppress AR expression by enhancing NCL binding to the G4-element in the *AR* promoter, two commercially available known G4-interactive compounds, the porphyrin TMPyP4 [34] and the quindoline derivatitive, SYUIQ-05 [35] (Supplementary Figure 4A), were tested for their capacity to suppress *AR* expression. In LNCaP and C4-2 cells treated for 24 hours with different concentrations of these two G4-binding compounds, TMPyP4 suppressed both AR mRNA and protein expression, whereas SYUIQ-05 increased AR mRNA and protein expression (Figure 4A and Figure 4B). TMPyP4 had no effect on NCL protein expression or cell viability at the tested concentrations (Figure 4A and Supplementary Figure 4B). A decrease in NCL expression was observed at 6 μM SYUIQ-05, but at this concentration, a decrease of 40% cell viability in LNCaP and VCaP was also observed after 24 h treatment (Supplementary Figure 4B).

The capacity of TMPyP4 and SYUIQ to regulate AR expression was dependent on the G4-element as determined by measuring the luciferase activity of the wild type versus the G4-deleted *AR* promoter reporter. TMPyP4 inhibited the wild type *AR* reporter over 4-fold, whereas SYUIQ-05 enhanced it 1.7-fold compared to vehicle treatment. However, deletion of the G4-element decreased the ability of TMPyP4 and SYUIQ-05 to affect the *AR* reporter (Figure 4C).

We hypothesized that the opposing activity of these G4-binding compounds on *AR* expression might be caused by their differential ability to affect NCL binding to the G4-element of the *AR* promoter. To test this, we performed NCL ChIP assays in LNCaP cells treated with 25 μM TMPyP4 and 3μM SYUIQ-05. qRT-PCR analysis of DNA retrieved from NCL ChIP showed that TMPyP4 increased, while SYUIQ-05 decreased the amount of NCL bound to the G4-element of the AR promoter, compared to control cells treated with DMSO (Figure 4D). These data indicate that G4-binding agents can modulate NCL association at the G4-element of the *AR* promoter and suggest that G4-binding compounds that increase NCL association with the G4 will inhibit *AR* gene transcription.

### Identification of new G4-binding drugs that suppress AR expression

**Figure 5:**
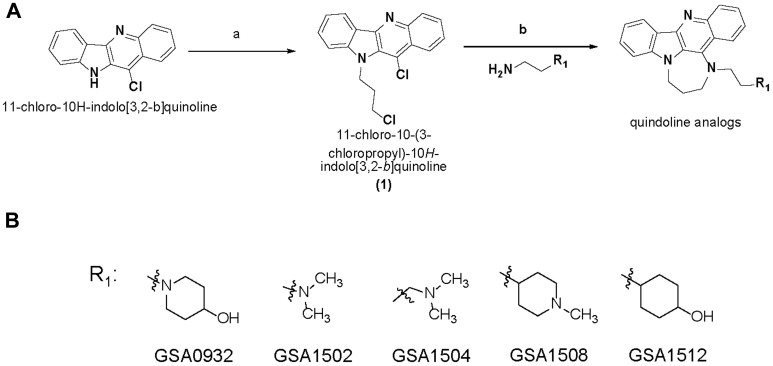
New G4-binding compound structures. (**A**) Synthesis of quindoline analogs. Reagents and conditions: (a) Sodium hydride, 1-bromo-3-chloropropane, DMF, 0° C-Room Temperature; (b) Neat, 100° C, sodium Iodide, different chemical moieties for R. (**B**) Chemical structure of GSA derivatives of quindoline.

**Figure 6:**
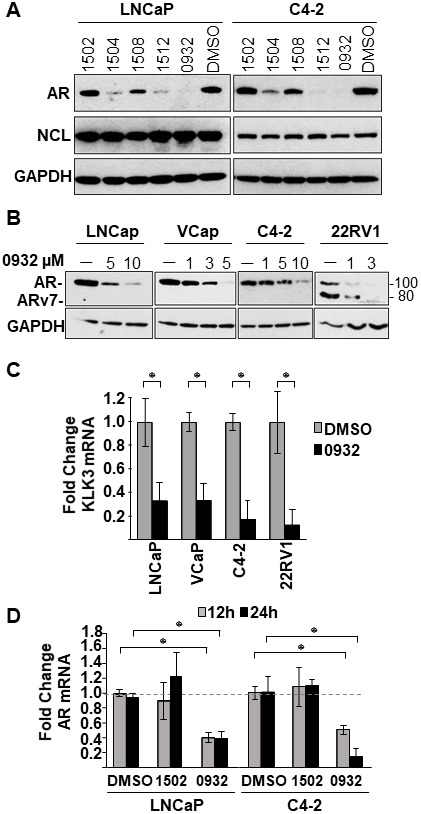
Effect of GSA derivatives on AR expression. (**A**) Cell lysates from LNCaP and C4-2 cells treated with indicated GSA compounds at a concentration of 10 μM for 24 hours were analyzed for expression of AR, NCL, and GAPDH by immunoblotting. (**B**) Cell lysates from indicated prostate cancer cell lines treated with increasing concentrations GSA0932 for 24 hours were analyzed for AR, NCL, and GAPDH by immunoblotting. (**C**) Extracted RNA from indicated prostate cancer cell lines treated for 12 hours with DMSO or GSA0932 (10 μM (LNCaP and C4-2), 5 μM (VCaP), or 3 μM (22RV1) was analyzed for expression of KLK3 (PSA) by RT-qPCR. (**D**) Extracted RNA from LNCaP or C4-2 cells treated for 12 or 24 hours with DMSO, 10 μM GSA0932, or 10 μM GSA1502 was analyzed for AR expression by RT-qPCR. Values are means ± SD; *p <* 0.05 (^*^); *n =* 3.

SYUIQ-05 and TMPyP4 suffer from high cytotoxicity or do not possess drug-like properties based on Lipinski’s rule of five [36], making them unsuitable for assessing biological functions or clinical development. Therefore, to identify less toxic compounds with drug-like properties and to further test our hypothesis that increased NCL association with the G4-element is required to inhibit *AR* gene transcription, an in-house proprietary library (GSA) of newly developed G4-binding drugs was screened. This new GSA class of small molecules, which are derivatives of a quindoline analog structure (Figure 5A), contain different chemical moieties for the R1 group (Figure 5B). Four of five members tested from this library suppressed AR protein expression (GSA0932> GSA1512> GSA1504> GSA1508) in androgen-dependent (LNCaP) and CRPC tumor cells (C2-4) after a 24h treatment at 10 μM (Figure 6A). GSA1502 did not affect AR protein expression, and none of these drugs affected the expression of NCL or GAPDH (Figure 6A). GSA0932 had the most potent inhibitory activity against AR, and its maximal inhibitory activity was observed at a concentration of 10 μM in both LNCaP and C4-2 cells (Figure 6B). Moreover, GSA0932 suppressed AR expression in 22RV1 and VCaP tumor cells after 24h of treatment, reaching its maximal inhibitory activity at a concentration of 3 and 5 μM respectively (Figure 6B). Importantly, GSA0932 also inhibited the expression of the clinically relevant *ARv7* splice variant in 22RV1 (Figure 6B) and suppressed mRNA expression of the classical AR target gene, *KLK3*, also known as PSA (Figure 6C). GSA0932, but not GSA1502, also significantly decreased AR mRNA in LNCaP and C4-2 cells after 12 and 24 hours of treatment (Figure 6D). Thus, we have identified new G4-binding drugs capable of transcriptionally inhibiting AR expression.

### AR suppression by GSA0932 requires the G4 element and NCL

**Figure 7:**
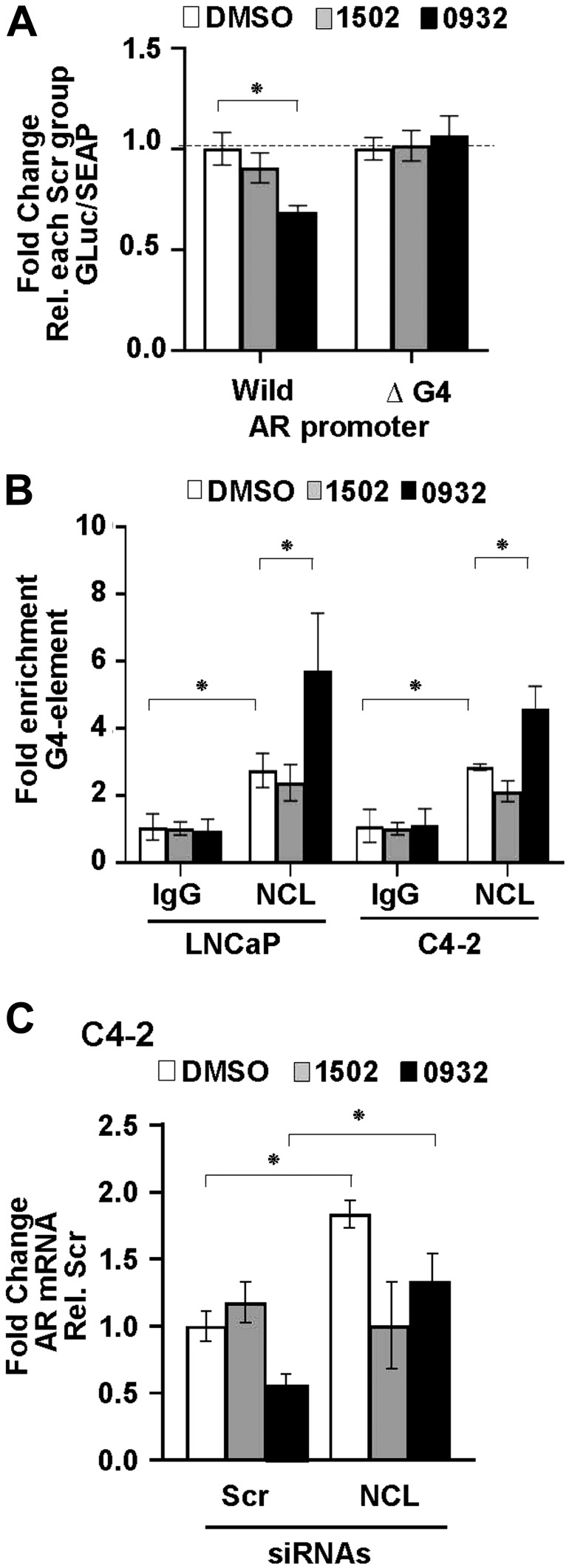
GSA0932 requires NCL binding to G4-element of *AR* to suppress AR. (**A**) Relative luciferase in LNCaP cells stably expressing the *AR* G4 (Wild) or deleted G4 (ΔG4) reporter, treated with DMSO, 10 μM GSA0932, or 10 μM GSA1502 for 12 hours. Luciferase activity was normalized to G-Luc/SEAP ratio and expressed as fold change relative to vehicle (DMSO). (**B**) ChIP of NCL on *AR* G4 in the absence or presence of 10 μM GSA0932. Negative (IgG) control. Plotted as fold enrichment relative to IgG. (**C**) LNCaP cells were transfected with scrambled (Scr) or NCL (NCL) siRNAs and 72 h post-transfection, cells were treated with DMSO, 10 μM GSA0932, or 10 μM GSA1502 for 12 hours. Extracted RNA was analyzed for AR expression by RT-qPCR. Values are means ± SD; *p* < 0.05 (^*^); *n =* 3.

To determine if GSA0932 suppresses *AR* via NCL binding to the G4, we first used our wild type and G4-deleted luciferase reporters. Relative to vehicle-treated control cells, GSA0932, but not GSA1502, significantly decreased luciferase activity of the wild type reporter (~40%, *p =* 00013) (Figure 7A). However, GSA0932 had no effect on the G4-deleted *AR* reporter (Figure 7A). Next, we assessed whether GSA0932 increases NCL association at the G4-element of the *AR* promoter using ChIP. GSA0932, but not GSA1502, increased the amount of NCL bound to the G4-element of the *AR* promoter by 2-fold in both LNCaP and C4-2 cells (Figure 7B). Moreover, knocking down NCL expression alleviated the GSA0932 inhibitory activity against *AR* mRNA expression compared with control cells (Figure 7C). Altogether, our findings demonstrate that the ability of G4-binding drugs to suppress AR expression requires that they increase NCL binding to the G4-element of the *AR* promoter.

### AR expression inhibitory activity of the G4-binding compounds is not related to their stabilization ability

**Figure 8:**
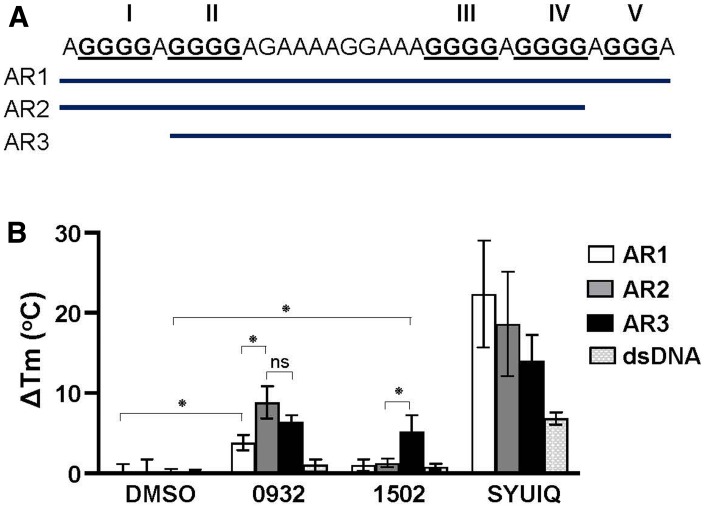
Stabilization capacity of G4-binding compounds for AR G4. (**A**) Representation of DNA oligonucleotide sequences (AR1, AR2, and AR3) from AR promoter that can form G4s. (**B**) ΔTm values obtained by CD spectroscopic melting analysis. AR1, AR2, and AR3 G4s (4 μM) in 100 mM K + were submitted to thermal melting unfolding in the absence (DMSO) or presence of TMPyP4, SYUIQ-05, GSA0932 and GSA1502 (16 μM). Thermal unfolding experiments were performed three times. Values are means ± SD; no significant difference (ns); *p* < 0.05 (^*^)

Circular dichroism thermal melting experiments were performed to investigate whether the effect of the G4-binding compounds on AR expression depends on their *AR* G4 stabilization capacity. The G-rich strand of the *AR* promoter is a 33-nt segment that contains five runs of guanine tracts (G-tract) (Figure 8A). Biophysical and computational studies have determined that these five putative G-tracts can contribute to the formation of multiple G4 structures using different G-tracts [18, 29]. Therefore, compounds that suppress (TMPyP4 and GSA0932), do not suppress (GSA1502), or activate (SYUIQ-05) AR expression were assessed for their ability to stabilize *AR* G4 structures formed by the three different combinations of *AR* promoter G-tracts — AR1 (I-V), AR2 (I-IV), and AR3 (II-V) (Figure 8A). The change in melting temperature (ΔT_m_) upon drug binding showed that these four G4-binding compounds were able to stabilize AR G4 structures (Figure 8B and Supplementary Figure 5). However, the compounds showed a differential capacity to stabilize *AR* G4 structures depending on the G-tract combination of the *AR* G4 structures. GSA0932 had a significant preference to stabilize the G4 structures formed by AR2 and AR3 sequences (ΔT_m_= 8.86° C and 6.45° C, respectively) over AR1 sequence (ΔT_m_= 3.83° C, AR1 vs. AR2 *p <* 0.01). In contrast, GSA 1502 only increased the stability of *AR* G4 structures formed by the AR3 sequence (ΔT_m_= 5.20 ° C) (Figure 8B and Supplementary Figure 5). GSA0932 and GSA1502 had no ability to stabilize dsDNA. TMPyP4 and SYUIQ-05 showed a higher capacity to stabilize *AR* G4 structures formed by any of the three *AR* sequences (Figure 8B and Supplementary Supplementary Figure 5A), but they also increased the stability of dsDNA (Figure 8B). TMPyP4 increased the T_m_ higher than 95° C in the three *AR* G4 sequences, making it impossible to determine the ΔT_m_ (Supplementary Figure 5B)_._ These results indicate that the stabilization capacity of the G4-binding compounds is not the sole determinant of their AR expression inhibitory activity.

GSA0932 was also able to stabilize the *c-Myc* G4 (data now shown) and block *c-Myc* mRNA expression (Supplementary Figure 6), indicating that GSA0932 is not necessarily specific for *AR* G4s. Interestingly, NCL binding to the *c-Myc* promoter G4 element has been reported to block *c-Myc* expression [26]. These data suggest that GSA0932 maybe have a preference for stabilizing the G4 isomer that is more efficient at NCL binding to maximally block *AR* gene transcription.

### GSA0932 selectively decreases the viability of AR-expressing prostate cancer cells

**Figure 9:**
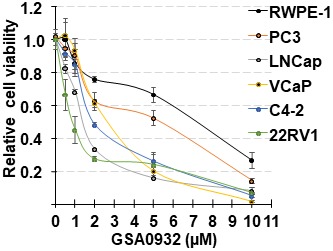
GSA0932 decreases cell viability. Indicated prostate cancer cell lines, or non-malignant prostate cells (RPWE), treated with different concentrations of GSA0932 for 48h and cell viability measured by MTT. Values are means ± SD; *n =* 3.

Because it is well-established that AR is critical for the growth of prostate cancer, and the loss of AR expression in tumor cells inhibits tumor growth [37–39], we determined whether GSA0932 affects tumor cell proliferation. AR-expressing prostate cancer cell lines, LNCaP, VCaP, C4-2, and 22RV1, non-AR expressing PC3 tumor cells, and non-tumorigenic RWPE-1 prostate epithelial cells were treated with varying concentrations of GSA0932 for 48 hours, and proliferation evaluated using the MTT assay. The resulting values were normalized to the proliferation of untreated cells for each cell line. GSA0932 has stronger cytotoxic activity against AR-positive tumor cells than non-AR expressing cells (Figure 9 and Table 1). Thus, GSA0932 is a new G4-stabilizing compound with potent inhibitory activity towards AR expression, displays AR-dependent selective cytotoxicity, and actively recruits NCL to the *AR* G4 to suppress *AR* gene transcription.

**Table 1:** IC_50_ of GSA0932

Cell line	IC50 (μM)
RWPE	5.4 ± 0.05
PC3	4.3 ± 0.16
LNCaP	1.4 ± 0.26
VCaP	2.8 ± 0.03
C4-2	2.0 ± 0.04
22Rv1	0.9 ± 0.11

## DISCUSSION

AR and its downstream signaling drive progression of both localized and advanced metastatic prostate cancer, making androgen deprivation therapy (ADT) the main initial treatment for patients with advanced PCa. These patients develop castration-resistant disease (CRPC), for which there are no curative therapies or significant advancements in treatment [2, 7]. Over 70% of CRPC tumors sustain a genetic alteration in the *AR* gene itself, such as mutation, amplification, altered splicing, or promoter/enhancer mutations that drive overexpression [40–42]. One common AR modification is the generation of altered splice variants that delete the androgen-binding domain [43]. The net effect of all the AR alterations is that the tumors remain dependent on AR, but no longer require physiological levels of androgen. Extensive research indicates that the ablation of AR expression might be an alternative strategy to develop a combinatory treatment for CRPC [37–39]. Approaches to block AR expression currently under investigation are to use RNAi (NCT02866916) or increase AR protein degradation [44, 45]. The findings in the present study support a new approach to block AR expression by targeting the G4 element in the *AR* promoter. Furthermore, we identified a specific molecular mechanism by which the G4-element within the *AR* promoter regulates its transcription. We demonstrate that NCL binds to the G4 element and is a transcriptional repressor of *AR*, and G4-binding compounds that block AR expression require NCL interaction with the G4 element.

NCL has a strong affinity for long looped-G4s [30, 31]. NCL affinity for G4 structures is directly correlated with the loop length [32], suggesting that the flexibility of the long loops in the G4s maybe produce an optimal fitting-ligand for NCL binding. Biophysical and computational studies of the G-rich sequence of the human *AR* promoter reveals that this sequence forms high flexible parallel G4 structures with a long central loop of 11 or 13 nucleotides [18, 29]. These studies also characterize the *AR* G4s as structures with low stability [29]. The low stability of *AR* G4 structures is also supported by studies showing that only the G4 structures with short loops are highly stable [46]. NCL also has the ability to bind and stimulate the folding of G-rich sequences that have the intrinsic characteristics to form unstable G4s [31]. Our observation that NCL binds to the G4-element of *AR* suggests that NCL is an essential protein to promote and protect the formation of a repressive G4 structure in the *AR* promoter to block its expression.

The findings in this study also indicate that NCL binding to *AR* G4 is essential to repress the transcriptional activity of the *AR* promoter. The G4-stabilizing compounds, TMPyP4 and GSA0932, which increase NCL association with the *AR* G4-element, show *AR* expression inhibitory activity depends on NCL expression. However, the G4-stabilizing compound SYUIQ-05, which induced *AR* expression, decreases NCL association with the *AR* G4-element. NCL binding to the *AR* G4 element might inhibit *AR* expression through different mechanisms such as blocking the transcription initiation complex and/or epigenetic remodeling of chromatin. The *AR* gene promoter lacks TATA and CCAAT boxes, and binding of Sp1 to the GC box at −60 to −50 bp, which lies downstream of G4-element at −118 to −75, drives transcription [12, 15, 47]. Thus, the NCL/G4 complex might affect the assembly of the transcription initiation machinery on the *AR* promoter. NCL increases the chromatin remodeling efficiency of the SWI/SNF machinery [48], suggesting that NCL may also facilitate the recruitment of SWI/SNF repressive complexes.

Further investigation will be required to resolve the structure of the GSA0932/*AR* G4 complex to determine how GSA0932 promotes NCL binding to *AR* G4. However, the findings in this study together with previously published studies provide some insights about the possible structural mechanism of GSA0932 binding to the *AR* G4 structure. The GSA compounds analyzed in this study are quindoline derivatives. Nuclear magnetic resonance studies of a quindoline derivative bond to a *c-Myc* G4, which takes a parallel-topology like the *AR* G4, revealed that the quindoline structure stacks over a total of three of the four guanines in the external G-tetrads [49]. Thus, it is very probable that the quindoline structure of GSA compounds binds to *AR* G4s in a similar fashion. The tested GSA compounds contain the same quindoline analog base structure and only differ in the formulation of the R1 group (Figure 5). Different chemical formulations of R1 change the *AR* expression repressor activity of the GSA compounds. SYUIQ-05 is also a quindoline derivative, but functions as an activator of AR expression by decreasing NCL binding to *AR* G4. SYUIQ-05 is formulated by the addition of a small R group (N, N-dimethyl-propane-1,3-diamine) to the quindoline structure without structural complexity (Supplementary Figure 4A). *AR* G4s are unstable, and the long loop makes them very flexible. Since NCL binding to G4s does not induce conformational changes in the G4 [32], it is likely, it is the R1 group in the GSA compound that is responsible for establishing the optimal *AR* G4 structure that permits efficient NCL binding and subsequent *AR* suppression. The chemical formulation of the R1 group in the GSA compounds would be predicted to flank specific bases in the DNA phosphate backbone in the grooves and/or in the long loop of *AR* G4 structure to stabilize the *AR* G4 conformation that makes an optimal fit between *AR* G4 structure and NCL.

The concept that R groups in quindoline structure impact the selectivity and stability capacity of the quindoline derivatives to specific G4 structures is supported by previous studies [50–52]. Thus, R groups with appropriate chemical formulation added to our quindoline analog base structure might lead to GSA compounds with selectivity for *AR* G4 and better AR expression suppressive activity by increasing NCL binding to *AR* promoter G4.

Recently, it has been reported that end stacking G4-binding compounds with high affinity and stabilization capacity such as PhenDC3 compete for the site of interaction of NCL in DNA G4, preventing NCL association [32]. Our findings suggest that the stabilizing capacity of the G4-binding compounds is not an indicator of NCL association to the *AR* promoter G4. TMPyP4 had a stronger stabilization capacity than SYUIQ-05. However, while TMPyP4 increases, SYUIQ-05 decreases the NCL binding to *AR* promoter G4. These observations also indicate that just the formation of the G4 structure in the *AR* promoter is not enough to repress the *AR* promoter activity, but also require the association of NCL.

NCL oncogenic functions have been extensively studied because its over-accumulation in the cytoplasm, mainly observed in cancer cells, regulates the expression of pro-survival or pro-apoptotic genes that promote cancer cell survival [53–56]. In the cytoplasmic, NCL RNA-binding activity regulated by its RNA binding domains and/or glycine/arginine-rich domain increases stability and translation of mRNA, enhancing polysome formation on the transcript [57–60]. However, NCL also has tumor suppressor activities [26, 61, 62], but these functions have been scarcely explored. Our studies suggest that NCL suppresses the oncogenic functions of AR by suppressing its expression via its interaction with the *AR* G4. In addition, NCL also suppresses the transcription of the *c-Myc* oncogene by its association with the G4 in the *c-MYC* promoter [26]. Therefore, these observations support that one of the mechanisms by which NCL functions as a tumor suppressor is through binding to the G4-elements of oncogene promoters to block their transcription.

Previous studies have also identified other molecules that stabilize the *AR* G4 and decrease the expression of AR [18, 19]. This study provides additional molecular insight into how G4 represses AR expression and suggests that the structural dynamic between NCL and the *AR* G4 fine tunes *AR* gene transcription. The deregulation of this mechanism might lead to enhanced PCa pathogenesis, particularly in CRPC. Our observations also reflect the combined importance of defining the cellular states that promote the formation of a NCL/G4 complex in the *AR* promoter and the molecular mechanism by which NCL at the *AR* G4 regulates *AR* expression in physiological and pathological conditions. A better understanding of NCL/*AR* G4 complex will be essential in the development of target-specific drugs that inhibit transcription of the *AR* gene.

## MATERIALS AND METHODS

### Cell culture and treatments

Cell lines were purchased from the American Type Culture Collection (ATCC) and routinely verified by short tandem repeat (STR) analysis every six months. Cells were used within 30 passages upon receipt from ATCC. Cells were grown in the appropriate medium supplemented with 10% fetal bovine serum (FBS, Hyclone Laboratories, Logan, Utah), 62.5 μg/mL penicillin, and 100 μg/mL streptomycin (Thermo Fisher Scientific) in a humidified incubator at 37° C with 5% CO_2_. RPMI was used for LCaP, C4-2, and 22RV1 cells. DMEM medium was used for VCaP cells. F-12K medium was used for PC3. Keratinocyte serum-free K-SFM medium supplemented with bovine pituitary extract and EGF (Thermo Fisher Scientific) was used for RWPE-1 cells. Cells were plated and incubated for 30 hours to allow adherence. Cells at 60% confluence were treated by direct addition of G4-binding compounds dissolved in DMSO (ATCC) to the supplemented culture medium to give the final concentration and time indicated in the figure legends. TMPyP4 (613560) and SYUIQ-5 (S5826) were purchased from Millipore-Sigma. Final DMSO concentrations in the cell media were < 0.1% in both vehicle control and G4-binding compound treatments.

### Chromatin immunoprecipitation

Chromatin immunoprecipitation (ChIP) assays were performed by using the chromatin immunoprecipitation kit from Cell Signaling Technology (CST #56383) following the manufacturer’s instructions. Briefly, cells (4.0 × 10^6^) were fixed in 0.9% formaldehyde (Thermo Fisher Scientific #56383) for 10 min. Fixation was stopped by adding glycine to the media to a final concentration of 125 mM and washed 3 with ice-cold PBS. Cells were scraped and pelleted at 1000 g for 10 min at 4° C. Nuclei were isolated by performing two consecutive resuspensions of the cell pellet in sonication cell lysis buffer (CST #96529) supplemented with protease and phosphatase inhibitor cocktails (Millipore Sigma). Nuclei were resuspended in sonication nuclear Lysis Buffer (CST #28778) supplemented with protease and phosphatase inhibitors. Chromatin was sheared into 200–500 bp with a BioRuptor^®^ Pico sonication device (Diagenode) at a sonication intensity for 30s on/60s off for 15 cycles at 4° C. DNA length was confirmed by agarose gel electrophoresis and ethidium bromide staining. Chromatin (20 μg) was immunoprecipitated with the appropriate antibody by overnight incubation at 4° C (Supplementary Table 1), followed by incubation with 30 μl of Dynabeads Protein G Magnetic Beads (Thermo Fisher Scientific) for 2 h at 4 ° C. The chromatin/antibody elution from the beads, the DNA cross-links reversion, and DNA purification were performed as recommended by the manufacturer (CST #56383). Purified DNA was analyzed by PCR-based amplification. For end-PCR, DNA was amplificated using the Phire Green Hot Start II PCR Master Mix (Thermo Fisher Scientific) with the following cycling conditions: an initial denaturation at 98° C for 3 min followed by 35 cycles at 98 ° C for 10 s, 62° C for 10 s and 72° C for 20 s. PCR products were separated by electrophoresis through 2% agarose gels and visualized by ethidium bromide intercalation. For qRT-PCR, DNA was amplified using Power select SYBR^®^ Green (Thermo Fisher Scientific). The primers used for ChIP PCR are shown in Supplementary Table 1.

### mRNA expression

Total RNA was isolated using the RNeasy Plus Kit (Qiagen). RNA (2 μg) was employed for cDNA synthesis using high-capacity cDNA reverse transcription Kit (Thermo Fisher Scientific). The resulting cDNAs (30 ng) were used as templates for qRT-PCR to analyze mRNA expression using Power select SYBR^®^ Green PCR Master Mix and primers for AR, NCL, KLK2, and PSA. Data were standardized to 18S plus GAPDH and were normalized (ΔΔCT). Primers were synthesized by Integrated DNA. The specific sequences are indicated in Supplementary Table 2.

### Cell extracts

Cells were lysed in cell lysis buffer [150 mmol/L NaCl, 2 mmol/L EDTA, 50 mmol/L Tris-HCl, 0.25% deoxycholic acid, 1% IGEPAL CA-630 (pH 7.5)] containing protease and phosphatase inhibitor cocktails (Millipore Sigma) for 5 minutes at 4° C and then cleared by centrifugation at 16,000 × g for 10 minutes at 4° C. All protein concentrations were determined using the bicinchoninic acid assay (Thermo Fisher Scientific).

### siRNA transfections

siRNA sequences against NCL, 5′-GGCAAAGCAUUGGUAGCAAtt-3′ (NCL1), and 5′-CGGUGAAAUUGAUGGAAAUtt-3′ (NCL2) were chemically synthesized and annealed by Ambion, Inc. BLAST analysis showed no homology of the siRNA sequences to any other sequences in the Human Genome Database. The siRNAs were transfected using Lipofectamine™ RNAiMAX (Thermo Fisher Scientific) according to the manufacturer's directions. Scrambled siRNA used as a negative control was from Ambion.

### Immunoblotting

Total cell lysates were resolved by SDS-Tris PAGE and transferred onto polyvinylidene fluoride membranes (Thermo Fisher Scientific) in Tris-glycine buffer containing 20% methanol. Proteins were detected by immunoblotting with an appropriate antibody overnight at 4° C (Supplementary Table 3). Membranes were stripped of bound antibodies using 62.5 mmol/L Tris-HCl (pH 6.7), 100 mmol/L 2-mercaptoethanol, and 2% SDS for 30 minutes at 60° C and reprobed as detailed in figure legends.

### AR promoter dual-reporter constructs

Two Gaussia luciferase/secreted alkaline phosphatase (GLuc/SEAP) dual-reporter systems were generated using the pEZX-PG04 vectors from GeneCopoeia. The GLuc gene was driven either by the wild type (WT) or mutant *AR* proximal promoter sequence. Wild AR promoter reporter contains the DNA segment spanning -1075 to +304 of the AR promoter (NC_000023.11; 67,542,957 to 67,544) [47]. The mutant AR promoter (ΔG4) contains the same sequence, except 33 bases that can form G-quadruplex structures (GGGGAGGGGAGAAAAGGAAAGGGGAGGGGAGGG) was deleted [18, 29]. In the same pEZX-PG04 vector, the SEAP gene is driven by a cytomegalovirus (CMV) promoter and serves as the internal control for signal normalization. The non-promoter luciferase reporter was used as a negative control to detect the basal activity of the dual-reporter vector. The constructs were confirmed by DNA sequencing (Eton Biosciences, San Diego, CA).

### Luciferase assay

LNCaP cells were transfected with each AR promoter dual-reporter or empty vector using Lipofectamine 3000 (ThermoFisher Scientific). Cells were incubated under standard conditions for two days before puromycin selection. The activities of GLuc and SEAP released from a stable pool into the culture medium were determined using the Secrete-Pair™ Dual Luminescence and Gaussia Luciferase Assay Kit (GeneCpoiea). The luminescence was measured using a PerkinElmer Enspire 2300 Multilabel Reader. The signal from the medium of cells expressing the empty vector was subtracted as background. Gaussia luciferase activity within each sample was double normalized with SEAP and cell number (measured by a 3-(4,5-dimethylthiazol-2-yl)-2,5-diphenyltetrazolium bromide (MTT) assay immediately after measuring GLuc and SEAP activity).

### Synthesis of GSA quindoline analogs

All solvents and reagents were purchased from commercial sources and were of the highest grade available unless otherwise noted. Flash chromatography was performed with silica gel (230/400 mesh, Fisher Scientific) on Biotage Sp1 purification system. All anhydrous reactions were carried out under positive pressure of nitrogen or argon. All microwave reactions were carried out on Biotage microwave initiator 2.5 system with power range 0-400 W at 2.45 GHz. HPLC-MS analyses were performed on Agilent 1100 series instrument with Zorbax C18 reverse phase column unless otherwise noted. HRMS results were obtained on an apex-Qe instrument. All ^1^H-NMR and ^13^C-NMR spectra were recorded on a Bruker 300 MHz or DRX 500 MHz NMR spectrometer, using deuterated solvents. The spectra are reported in ppm and referenced to deuterated DMSO (2.49 ppm for ^1^H, 39.5 ppm for ^13^C) or referenced to deuterated chloroform (7.26 ppm for ^1^H, 77 ppm for ^13^C).

### Synthesis of 10-(3-chloropropyl)-11-chloro-10H-indolo[3,2-b] quinoline (1)

To a stirred solution of 11-chloro-10H-indolo[3,2-b] quinoline (8.0 g) in dry DMF (80 mL), 60% sodium hydride in mineral oil (4.3 g) was added at 0 ° C. The reaction mixture was stirred at the same temperature for 1 hour and 1,3-bromochloropropane (22.8 g) was added drop wise to the reaction mixture. The reaction mixture was poured into ice cold water and extracted with EtOAc (3 × 100 mL). The combined EtOAc layers were washed with water, and concentrated to obtain the crude product. The crude product was purified by silica gel column chromatography using 2-4% EtOAc in hexane as an eluent to afford 6.0 g (57%) of 10-(3-chloropropyl)-11-chloro-*10H*-indolo[3,2–*b*] quinoline as yellow solid. ^1^H NMR (300 MHz, CDCl_3_): δ 8.54 (d, *J* = 7.5 Hz, 1H, ArH), 8.43 (d, *J* = 8.1 Hz, 1H, ArH), 8.34 (d, *J* = 8.4 Hz, 1H, ArH), 7.80 - 7.60 (m, 3H, ArH), 7.59 (d, *J* = 8.4 Hz, 1H, ArH), 7.40 (t, *J* = 7.3 Hz, 1H, ArH), 4.95 (t, *J* = 7.2 Hz, 2H), 3.67 (t, *J* = 6.1 Hz, 2H), 2.50 - 2.40 (m, 2H). MS (ESI): *m/z* 329.2 and 331.2 (M+H)^+^].

These quindoline analogs were synthesized from 11-chloroquindoline, as shown in scheme 1. Structures, chemical nomenclature, and analytical data on tested compounds are shown in Supplementary Table 4.

### Synthesis of GSA0932 and its dihydrochloride salt

A mixture of 10-(3-chloropropyl)-11-chloro-10H-indolo[3,2–b] quinoline (1 g, 3.04 mmol), sodium iodide (1.1 g, 7.3 mmol) and N-(2-aminoethyl)-4-piperidinol (2.0 g, 13.88 mmol) was stirred at 100 ° C for 24 h. The progress of the reaction was monitored by TLC (10% MeOH in CHCl_3_). After completion, the reaction mixture was poured on to ice-cold water and extracted with EtOAc (3 × 100 mL). The combined EtOAc layers were washed with water (3 × 200 mL) and concentrated under reduced pressure to give the crude product. The crude product was purified by silica gel column chromatography using 2–4% MeOH in CHCl_3_ as eluent to afford 1.0 g (84%) of pure product as yellow color solid. This product was converted into dihydrochloride salt using methanolic HCl (3 N solution, 8.5 mL, 10 mmol) at 0 ° C and then room temperature. Diethyl ether (50 mL) was added to the reaction mixture, and the resulting solids were collected by filtration. The product was recrystallized from a mixture of solvents (methanol, DCM, and ether) to yield 1.1 g of pure CV-GSA-02/30 as yellow color solid in 93% yield (Supplementary Table 4).

### Synthesis of GSA1502, GSA1504, GSA1508, and GSA1512

These compounds were synthesized using microwave irradiation at 130 ° C for 25 min. The reaction mixture was poured into water and extracted with dichloromethane (100 mL × 3). The combined organic layer was washed, dried, filtered, and concentrated. The resulting residue was purified on silica gel column to obtain pure compounds. Reactions details are discussed below.

### Synthesis of GSA1502

A mixture of 11-chloro-10-(3-chloropropyl)-10H-indolo[3,2-b] quinoline (0.3 g, 1.23 mmol), N1, N1-dimethylethane-1,2-diamine (2 mL, excess) and NaI (404 mg, 2.70 mmol) were reacted under microwave irradiation at 130 ° C for 25 min. After completion of the reaction, the reaction mixture was poured into water and extracted with dichloromethane (100 mL × 3). The combined organic layer was washed, dried, filtered, and concentrated. The resulting residue was purified on silicone gel column to give compound 25 mg as yellow solid (Supplementary Table 4).

### Synthesis of GSA1504

A mixture of 11-chloro-10-(3-chloropropyl)-10H-indolo[3,2-b] quinoline (0.3 g, 1.23 mmol), N1, N1-dimethylpropane-1,2-diamine (2 mL, excess) and NaI (404 mg, 2.70 mmol) were reacted to give compound 100 mg as yellow solid (Supplementary Table 4).

### Synthesis of GSA1508

A solution of 11-chloro-10-(3-chloropropyl)-10H-indolo[3,2-b] quinolone (200 mg, 0.6 mmol), 2-(1-methylpiperidin-4-yl) ethan-1-amine (261.0 mg, 1.8 mmol) and NaI (91.0 mg, 0.6 mmol) in 2 mL DMF were reacted to obtain compound 52 mg as yellow solid (Supplementary Table 4).

### Synthesis of GSA1512

A solution of 11-chloro-10-(3-chloropropyl)-10H-indolo[3,2-b] quinolone (200 mg, 0.6 mmol), 4-(2-aminoethyl) cyclohexan-1-ol (261.0 mg, 1.8 mmol) and NaI (91.0 mg, 0.6 mmol) in 2 mL DMF were reacted to obtain 50 mg of GSA1512 as yellow solid (Supplementary Table 4).

### Circular dichroism spectroscopy

*AR* G4 oligonucleotides (Supplementary Table 5) were diluted to 4 μM in 10 mM sodium cacodylate pH 7.4 buffer containing 100 mM KCl without (DMSO) or with 4 equivalents (16 μM) of GSA 0932, GSA 1502, SYUIQ-5, or TMPyP4. For dsDNA, equimolar amounts of ssDNA and its complementary sequence were used (Supplementary Table 5). The samples were annealed by heating to 95° C for 1 min and an additional 1 min after adding compounds. The samples were cooled down slowly. Melting curves were recorded at a proper wavelength with increasing temperatures from 20 to 95° C at a rate of 1° C/min on a JASCO-810 spectropolarimeter using 1mm path length quartz cuvettes, 1nm bandwidth, and 1s of response time.

### Cell viability assays

Cell viability was indirectly examined using a 3-(4,5-dimethylthiazol-2-yl)-2,5-diphenyltetrazolium bromide (MTT) assay [63]. Briefly, 8000 cells were seeded in quadruplicate in 96-well plates and allowed to adhere for 24 hours. Cells were treated with different concentrations of GSA0932 and incubated for 48 hours without changing the culture medium. After treatment, cell viability was measured using MTT. The signal corresponding to medium with no cells was subtracted as background. Cell proliferation was determined by normalizing to the proliferation of untreated cells for each cell type.

## SUPPLEMENTARY MATERIALS



## Abbreviations

AR: androgen receptor
ADT: androgen-deprivation therapies
CRPC: castration-resistant prostate cancer
NCL: nucleolin
G4: G-quadruplex
Sp1: specific protein 1
TIS: transcription initiation site
ChIP: chromatin immunoprecipitation
