# Correction: Nucleolin represses transcription of the androgen receptor gene through a G-quadruplex

**DOI:** 10.18632/oncotarget.27661

**Published:** 2020-06-30

**Authors:** Cindy K. Miranti, Sara Moore, Yongeun Kim, Venkateshwar Reddy Chappeta, Kui Wu, Biswanath De, Vijay Gokhale, Laurence H. Hurley, Elsa M. Reyes-Reyes

**Affiliations:** ^1^ University of Arizona Cancer Center, University of Arizona, Tucson, AZ 85724, USA; ^2^ Department of Cellular and Molecular Medicine, Tucson, AZ 85724, USA; ^3^ University of Arizona College of Medicine, Division of Pulmonary, Allergy, Critical Care, and Sleep Medicine, Tucson, AZ 85724, USA; ^4^ College of Pharmacy, University of Arizona, Tucson, AZ 85724, USA; ^5^ BIO5 Institute, University of Arizona, Tucson, AZ 85724, USA

**This article has been corrected:** The Acknowledgements section has been updated as follows:

## ACKNOWLEDGMENTS

These studies were supported by funds from IRG-16-124-37 from the America Cancer Society and University of Arizona Career Development Award to EMRR and by NIH award R01CA177585 to L.H.H.

Original article: Oncotarget. 2020; 11:1758–1776. 1758-1776. https://doi.org/10.18632/oncotarget.27589

