# Supplementary material for: Nucleolin represses transcription of the androgen receptor gene through a G-quadruplex
Source: Oncotarget. 2020 May 12;11(19):1758–76. doi: 10.18632/oncotarget.27589 (PMC7233804; doi:10.18632/oncotarget.27589)
Supplement: Supplementary file 1 [file oncotarget-11-1758-s001.pdf]

Nucleolin represses transcription of the androgen receptor gene through a G-quadruplex

SUPPLEMENTARY MATERIALS

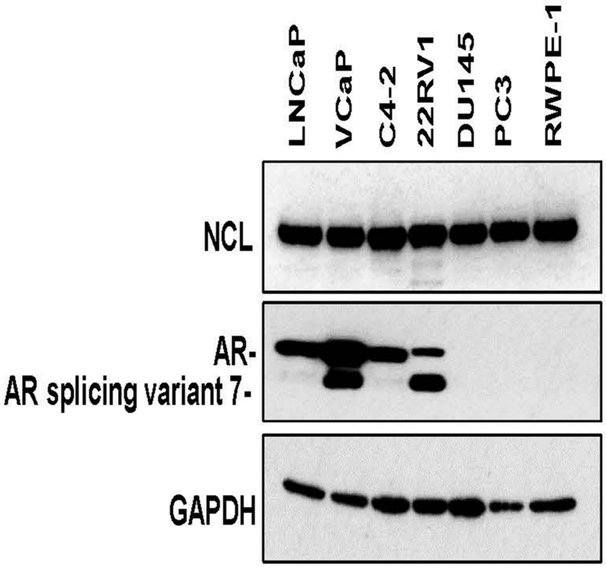

Supplementary Figure 1: NCL and AR expression in different prostate cell lines. Prostate cell lysates were analyzed for expression of NCL and GAPDH by immunoblotting.

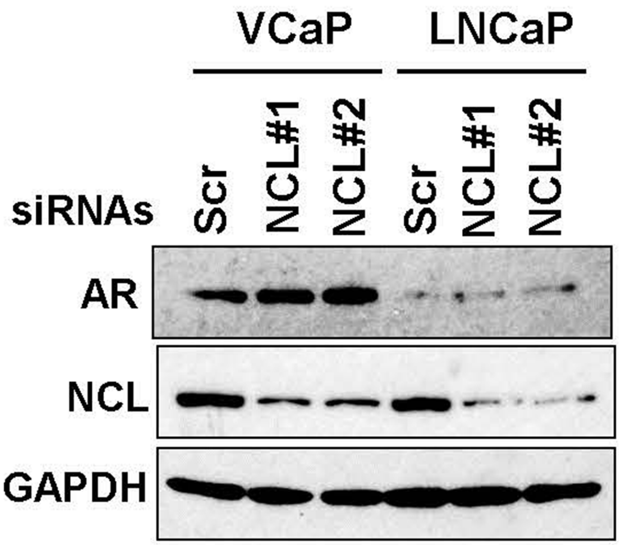

Supplementary Figure 2: NCL suppresses AR expression. Indicated prostate cancer cell lines were transfected with NCL (NCL#1,#2) or scramble (Scr, control) siRNAs. Cell lysates were analyzed for expression of AR, NCL, and GAPDH by immunoblotting.

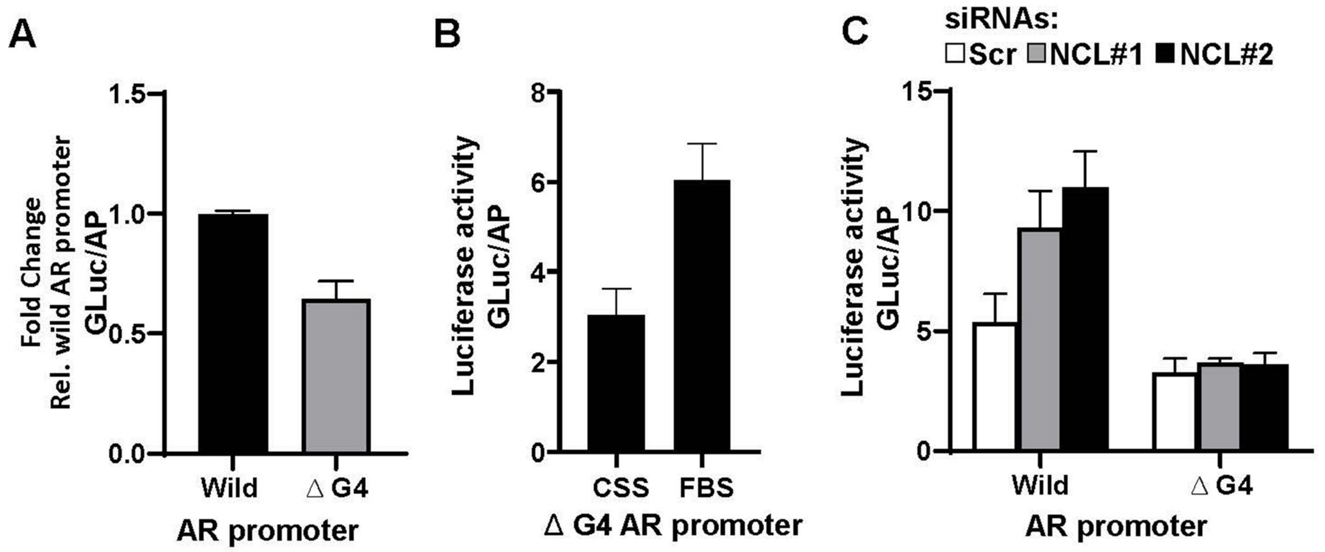

Supplementary Figure 3: Activity of wild or mutant (deleted G4 sequence (ΔG4)) AR promoter. (**A**) Measure of the basal luciferase activity of the wild type and ΔG4 AR promoter dual-luciferase/SEAP reporters stably expressed in LNCaP. Fold luciferase activity relative to wild AR promoter after normalization to G-Luc/SEAP ratio. (**B**) LNCaP cells stably expressing the ΔG4 AR promoter dual-luciferase/SEAP reporter were growth in phenol red-free media supplemented with 1% charcoal-stripped serum (CSS) for 18 h. Then, cells were incubated with fresh cell media supplemented with 1% CSS or 10% full serum (FBS) for 8 hr. (**C**) LNCaP cells stably expressing the wild type or ΔG4 AR promoter dual-luciferase/SEAP reporter were transfected with scrambled (Scr) or two NCL siRNAs (#1, #2). Luciferase activity normalized to G-Luc/SEAP ratio. Results from Fig. 3B before calculating fold change. Values are means ± SD; n=3.

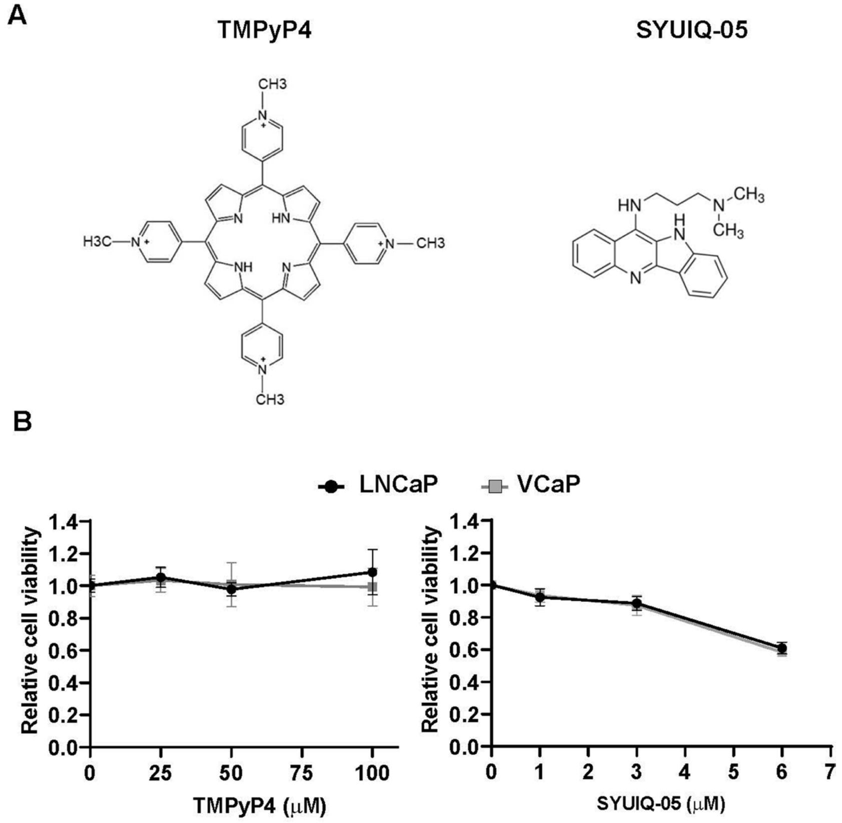

Supplementary Figure 4: Effect of TMPyP4 and SYUIQ-05 on cell viability. (**A**) Chemical structure of TMPyP4 and SYUIQ-05. (**B**) LNCaP and VCaP treated with different concentrations of TMPyP4 and SYUIQ-05 for 24h and cell viability measured by MTT. Values are means ± SD; n=3.

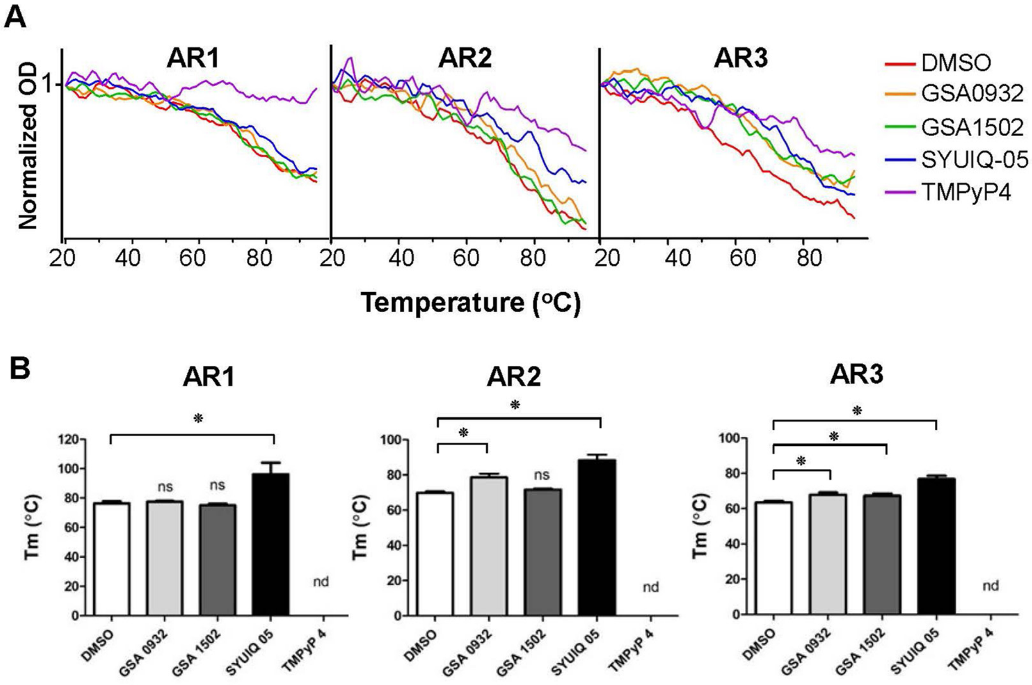

Supplementary Figure 5: CD spectroscopic melting experiments. AR1, AR2, and AR3 G4s (4 μM) 100 mM K+ were submitted to thermal melting in absence (DMSO) or presence of TMPyP4, SYUIQ-05, GSA0932, and GSA1502 (16 μM). (**A**) Representative spectroscopic tracing of the AR G4 melting curves. (**B**) Thermal stability bar graphs for AR G4s. Experiments were performed three times. Values are means ± SD; no significant difference (ns); $p < 0.05$ (*).

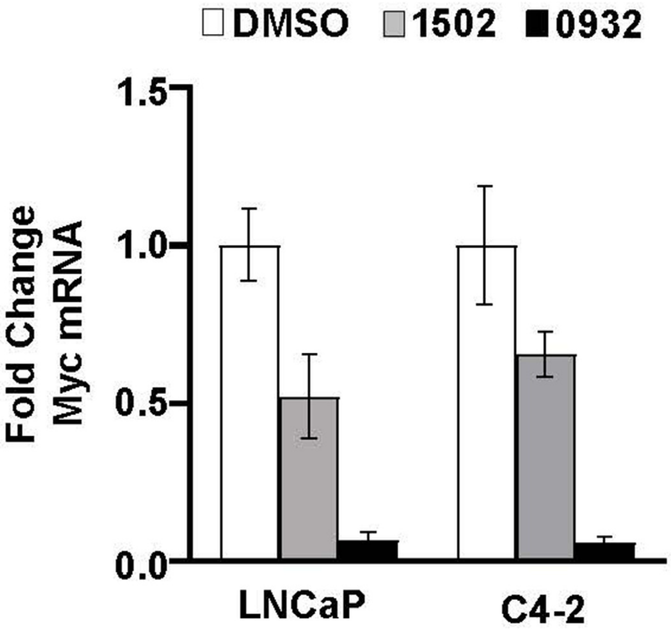

Supplementary Figure 6: Effect of GSA0932 and GSA1502 on c-Myc expression. Extracted RNA from indicated prostate cancer cell lines treated for 12 hours with DMSO or 10 μM GSA0932 or GSA1502 was analyzed for expression of c-Myc by RT-qPCR.

Supplementary Table 1: Primers for genomic element amplification

Genomic Region		Sequence (5'→ 3')
G4	Fwd	TATTCAGGAAGCAGGGGTCC
(160 bp)	Rev	CTTCCCACCTCCTTTTCCCT
Exon 1	Fwd	CAACTCCTTCAGCAACAGCA
(160 bp)	Rev	CCGACACTGCCTTACACAAC

Supplementary Table 2: qRT-PCR primers for measure mRNA expression

Gene		Sequence (5'→ 3')
AR	Fwd	CCTGGCTTCCGCAACTTACAC
	Rev	GGACTTGTGCATGCGGTACTCA
NCL	Fwd	AGCTTCTGTCCCTCTGCTTT
	Rev	TTCCAAGGAGACCACAGGAC
PSA	Fwd	AAAAGCGTGATCTTGCTGGG
(KLK3)	Rev	TCACAGCATCCGTGAGCTC
KLK2	Fwd	CGAACCAGAGGAGTTCTTGCG
	Rev	CAGGGCACATGGCTCAGGG
GAPDH	Fwd	GATCATCAGCAATGCCTCCTGC
	Rev	CTTCTGGGTGGCAGTGATGGC
cMyc	Fwd	TTCGGGTAGTGGAAAACCAG
	Rev	AGTAGAAATACGGCTGCACC

Supplementary Table 3: Antibodies

Target	Species	Mono/Poly	Company	Product no.	Application
NCL	mouse	mono	Santa Cruz	sc-55486	ChIP
Histone 3	mouse	mono	Abcam	10799	ChIP
Histone 3	rabbit	mono	CST	4620	ChIP
IgG	mouse	normal Isotype	CST	5415	ChIP
IgG	rabbit	normal Isotype	CST	2729	ChIP
NCL	mouse	mono	Santa Cruz	sc-8031	WB
Androgen Receptor	mouse	mono	Santa Cruz	sc-7305	WB
Flag M2	mouse	mono	Sigma	F1804	WB
GAPDH	mouse	mono	Santa Cruz	Sc-47724	WB
horseradish peroxidase mouse IgG	Horse	poly	CTS	7076	WB

Supplementary Table 4: Analytical data of tested quindoline analogs

Structure/Name	Chemistry Analytical Data
GSA0932<br>1,2,3,4-tetrahydro-1-[4-hydroxypiperidinyl-1-ethyl][1,4] diazepino[1,2,3-no]quindoline	^{1}H NMR (D_2O, 500 MHz): d 7.66 (d, J = 8.5 Hz, 1H, ArH), 7.55 (t, J = 7.7 Hz, 1H, ArH), 7.46 (d, J = 8.5 Hz, 1H, ArH), 7.42-7.34 (m, 2H, ArH), 7.04 (t, J = 7.7 Hz, 1H, ArH), 6.89 (d, J = 8.5 Hz, 1H, ArH), 6.67 (t, J = 7.2 Hz, 1H, ArH), 4.05-3.82 (m, 3H), 3.81-3.72 (m, 2H), 3.62-3.55 (m, 2H), 3.54-3.48 (m, 2H), 3.47-3.22 (m, 2H), 3.21-3.0 (m, 2H), 2.28-2.15 (m, 2H), 2.14-1.85 (m, 2H), 1.83-1.52 (m, 2H).<br>^{13}C NMR (D_2O, 250 MHz): 147.38, 143.58, 135.58, 134.35, 132.42, 132.36, 125.61, 125.34, 123.90, 121.40, 119.21, 117.91, 112.89, 111,11, 53.80, 52.49, 51.48, 47.16, 26.48.<br>HPLC Purity = 100 %<br>HRMS Found = 401.2341(MH^+) (Theoretically = 401.2336) Error = -1.5 ppm.
GSA1502<br>1,2,3,4-tetrahydro-1-[N,N-dimethylamino-1-ethyl][1,4] diazepino[1,2,3-no]quindoline	^{1}H NMR (400 MHz, $CDCl_3$) δ 8.54 (ddd, J = 7.7, 1.2, 0.7 Hz, 1H), 8.31 – 8.26 (m, 2H), 7.68 – 7.57 (m, 2H), 7.51 (ddd, J = 6.7, 4.4, 1.3 Hz, 1H), 7.41 (d, J = 8.2 Hz, 1H), 7.35 – 7.30 (m, 1H), 4.23 – 4.17 (m, 2H), 3.71 – 3.67 (m, 2H), 3.51 – 3.45 (m, 2H), 2.79 – 2.73 (m, 2H), 2.41 (ddd, J = 11.4, 5.7, 2.6 Hz, 2H), 2.28 (s, 6H).<br>^{13}C NMR (101 MHz, $CDCl_3$) δ 147.63, 145.99, 145.43, 139.04, 129.35, 129.28, 126.58, 123.94, 123.88, 123.03, 122.92, 121.99, 120.00, 109.68, 58.30, 52.55, 50.54, 46.64, 45.99, 25.88.<br>HPLC Purity = 100 %<br>HRMS: Found = 345.2072 (MH^+) (Theoretically = 344.2001) Error = -0.7 ppm.
GSA1504<br>(1,2,3,4-tetrahydro-1-[N,N-dimethylamino-1-propyl][1,4] diazepino[1,2,3-no]quindoline)	^{1}H NMR (400 MHz, $CDCl_3$) δ 8.54 (ddd, J = 7.8, 1.3, 0.7 Hz, 1H), 8.29 (ddd, J = 8.5, 1.3, 0.6 Hz, 1H), 8.23 (ddd, J = 8.5, 1.5, 0.6 Hz, 1H), 7.67 – 7.59 (m, 2H), 7.51 (ddd, J = 8.5, 6.7, 1.3 Hz, 1H), 7.44 (d, J = 8.3 Hz, 1H), 7.33 (ddd, J = 7.9, 7.2, 0.9 Hz, 1H), 4.25 – 4.19 (m, 2H), 3.74 – 3.68 (m, 2H), 3.40 (dd, J = 8.7, 6.7 Hz, 2H), 2.46 – 2.40 (m, 4H), 2.33 (s, 6H), 2.08 (tt, J = 9.9, 6.5 Hz, 2H).<br>^{13}C NMR (101 MHz, $CDCl_3$) δ 147.44, 145.82, 145.51, 139.25, 129.46, 129.34, 129.17, 126.68, 124.00, 123.88, 122.91, 122.08, 120.11, 109.77, 56.85, 52.41, 50.08, 46.73, 45.15, 26.53, 25.79.<br>HPLC Purity = 99. 8 %<br>HRMS: Found = 359.2230 (MH+) (Theoretically = 358.2157) Error = -0.6 ppm.
GSA1508<br>1,2,3,4-tetrahydro-1-[1-methyl-4-piperidinyl-1-ethyl][1,4] diazepino[1,2,3-no]quindoline	^{1}H NMR (400 MHz, $CDCl_3$) δ 8.50 – 8.46 (m, 1H), 8.25 – 8.21 (m, 1H), 8.10 (ddd, J = 8.5, 1.4, 0.5 Hz, 1H), 7.62 – 7.57 (m, 1H), 7.52 (ddd, J = 8.3, 5.2, 1.3 Hz, 1H), 7.43 (ddd, J = 8.5, 6.7, 1.3 Hz, 1H), 7.31 – 7.24 (m, 2H), 4.08 – 4.02 (m, 2H), 3.56 – 3.50 (m, 2H), 3.24 – 3.16 (m, 2H), 2.87 (d, J = 11.8 Hz, 2H), 2.31 – 2.20 (m, 5H including –CH_3), 1.94 (td, J = 11.9, 2.5 Hz, 2H), 1.78 – 1.69 (m, 2H), 1.60 (d, J = 12.7 Hz, 2H), 1.42 (ddd, J = 25.3, 12.6, 3.8 Hz, 2H), 1.24 – 1.12 (m, 1H).<br>^{13}C NMR (101 MHz, $CDCl_3$) δ 147.46, 145.90, 145.29, 139.31, 129.36, 129.26, 128.90, 126.56, 123.96, 123.82, 122.85, 122.86, 121.86, 119.95, 109.690, 55.43, 52.13, 49.76, 46.69, 45.79, 35.12, 32.86, 31.76, 25.55.<br>HPLC Purity = 93.5 %<br>HRMS: Found = 399.2543 (MH^+) (Theoretically = 399.2543) Error = 0.1 ppm.
GSA1512<br>1,2,3,4-tetrahydro-1-[4-hydroxycyclohexyl-1-ethyl][1,4] diazepino[1,2,3-no]quindoline	^{1}H NMR (400 MHz, $CDCl_3$) δ 8.55 (d, J = 7.3 Hz, 1H), 8.29 (dd, J = 8.5, 0.8 Hz, 1H), 8.21 (ddd, J = 8.5, 1.4, 0.5 Hz, 1H), 7.67 – 7.59 (m, 2H), 7.50 (ddd, J = 8.5, 6.7, 1.3 Hz, 1H), 7.43 (d, J = 8.3 Hz, 1H), 7.33 (ddd, J = 7.9, 7.2, 0.9 Hz, 1H), 4.25 – 4.19 (t, J = 0.8 Hz, 2H), 3.99 (dt, J = 5.3, 2.4 Hz, 1H), 3.71 – 3.65 (m, 2H), 3.38 – 3.31 (m, 2H), 2.45 – 2.36 (m, 2H), 1.89 – 1.81 (m, 2H), 1.73 (dd, J = 8.9, 4.9 Hz, 2H), 1.60 – 1.46 (m, 6H), 1.40 (dd, J = 14.8, 8.2 Hz, 1H).<br>^{13}C NMR (101 MHz, $CDCl_3$) δ 147.58, 145.98, 145.42, 139.61, 129.36, 129.29, 129.14, 126.60, 124.02, 123.85, 123.02, 122.08, 120.00, 109.67, 66.81, 52.58, 49.75, 46.81, 34.89, 34.42, 32.20, 27.20, 25.55.<br>HPLC Purity = 97.3 %<br>HRMS: Found = 400.2383 (MH+) (Theoretically = 400.2384) Error = -0.1 ppm.

Supplementary Table 5: Oligonucleotides for CD studies

Oligo	Sequence (5’→ 3’)
AR1	AGGGGAGGGGAGAAAAGGAAAGGGGAGGGGAGGGA
AR2	AGGGGAGGGGAGAAAAGGAAAGGGGAGGGG
AR3	AGGGGAGAAAAGGAAAGGGGAGGGGAGGGA
ssDNA	AGTGAGAGTGAGAGAGAGAGTGAGAGTGAGAGTGA
ssDNA complementary	TCACTCTCACTCTCACTCTCTCTCTCACTCTCACT
